# Cell-Cycle Inhibition by *Helicobacter pylori* L-Asparaginase

**DOI:** 10.1371/journal.pone.0013892

**Published:** 2010-11-09

**Authors:** Claudia Scotti, Patrizia Sommi, Maria Valentina Pasquetto, Donata Cappelletti, Simona Stivala, Paola Mignosi, Monica Savio, Laurent Roberto Chiarelli, Giovanna Valentini, Victor M. Bolanos-Garcia, Douglas Scott Merrell, Silvia Franchini, Maria Luisa Verona, Cristina Bolis, Enrico Solcia, Rachele Manca, Diego Franciotta, Andrea Casasco, Paola Filipazzi, Elisabetta Zardini, Vanio Vannini

**Affiliations:** 1 Department of Experimental Medicine, Section of General Pathology, University of Pavia, Pavia, Italy; 2 Section of Human Physiology, Department of Physiology, University of Pavia, Pavia, Italy; 3 Department of Biochemistry A. Castellani, University of Pavia, Pavia, Italy; 4 Department of Biochemistry, University of Cambridge, Cambridge, United Kingdom; 5 Department of Microbiology and Immunology, Uniformed Services University of the Health Sciences, Bethesda, Maryland, United States of America; 6 Department of Pharmaceutical Sciences, University of Modena and Reggio Emilia, Modena, Italy; 7 Centro Analisi Monza (CAM), Monza, Italy; 8 Department of Human Pathology, University of Pavia, and Pathologic Anatomy Service, Fondazione Istituto di Ricovero e Cura a Carattere Scientifico (IRCCS) Policlinico San Matteo, Pavia, Italy; 9 Laboratory of Neuroimmunology, Fondazione Istituto di Ricovero e Cura a Carattere Scientifico (IRCCS) Policlinico San Matteo, Neurological Institute C. Mondino, and University of Pavia, Pavia, Italy; 10 Department of Experimental Medicine, Section of Histology and General Embriology, University of Pavia, Pavia, Italy; 11 Unit of Immunotherapy of Human Tumours, Fondazione Istituto di Ricovero e Cura a Carattere Scientifico Istituto Nazionale Tumori, Milano, Italy; Max Planck Institute for Infection Biology, Germany

## Abstract

*Helicobacter pylori* (*H. pylori*) is a major human pathogen causing chronic gastritis, peptic ulcer, gastric cancer, and mucosa-associated lymphoid tissue lymphoma. One of the mechanisms whereby it induces damage depends on its interference with proliferation of host tissues. We here describe the discovery of a novel bacterial factor able to inhibit the cell-cycle of exposed cells, both of gastric and non-gastric origin. An integrated approach was adopted to isolate and characterise the molecule from the bacterial culture filtrate produced in a protein-free medium: size-exclusion chromatography, non-reducing gel electrophoresis, mass spectrometry, mutant analysis, recombinant protein expression and enzymatic assays. L-asparaginase was identified as the factor responsible for cell-cycle inhibition of fibroblasts and gastric cell lines. Its effect on cell-cycle was confirmed by inhibitors, a knockout strain and the action of recombinant L-asparaginase on cell lines. Interference with cell-cycle *in vitro* depended on cell genotype and was related to the expression levels of the concurrent enzyme asparagine synthetase. Bacterial subcellular distribution of L-asparaginase was also analysed along with its immunogenicity. *H. pylori* L-asparaginase is a novel antigen that functions as a cell-cycle inhibitor of fibroblasts and gastric cell lines. We give evidence supporting a role in the pathogenesis of *H. pylori*-related diseases and discuss its potential diagnostic application.

## Introduction

*Helicobacter pylori* is a common human pathogen that colonizes the gastric mucosa and induces DNA damage, chronic gastritis, peptic ulcer, gastric cancer, and mucosa-associated lymphoid tissue lymphoma of the stomach (class I carcinogen) [1]. Development of adenocarcinoma of the distal stomach in humans [2], [3] and in experimental animals [4], [5] has been associated with infection by the bacterium. There is experimental evidence demonstrating that stimulation of epithelial cell proliferation and increased apoptotic cell death [5]–[14] are among the pathogenetic processes involved in *H. pylori*'s associated diseases, which suggests that the bacterium could interfere with the maintenance of the integrity of the gastric mucosa and even favour tumour formation by affecting the normal balance between epithelial cell proliferation and cell death. *In vitro* studies where cultured cells were exposed to whole bacteria, bacterial lysates or broth culture filtrate, showed both increased [15] and decreased [16]–[20] cell proliferation, thus pointing to differential effects exerted by bacterial products on different cell lines.

In previous work we have shown that *H. pylori* bacterial broth culture filtrate (BCF) can induce cell-cycle arrest (G1 phase) in several cell lines in a vacuolating cytotoxin A (VacA), cytotoxin-associated gene A (CagA) and Urease-independent manner [21]. This evidence suggested that one or more unknown bacterial factors could have an important role in this process and prompted us to pursue its/their isolation.

The cell cycle of normal human fibroblasts (HDF) was particularly affected by BCF compared to other cell lines [21], thus they were selected as a reference model throughout this study. These cells are an interesting experimental system for several reasons: *in vivo* they are present in the subepithelial mucosa and can be a target of bacterial factors leaking through epithelial lesions or transferred there through the epithelium [22]; they participate in the immune response behaving as reactive tissue components, initiating the earliest molecular events of the inflammatory response [23], and also acting as antigen presenting cells. In this respect, we have previously demonstrated that interference with their function can potentially have profound consequences for immunobiology and tissue integrity both in the stomach and in areas other than the gastrointestinal tract [21].

Herein, we demonstrate that *H. pylori* L-asparaginase plays a major role in cell-cycle inhibition induced by BCF on cultured cells, that inhibition of proliferation is more pronounced in cells with low expression levels of the enzyme asparagine synthetase and that L-asparaginase can stimulate the immune response in infected patients.

## Results

### Identification of cell-cycle inhibiting activity in *H. pylori* BCF

**Figure 1:**
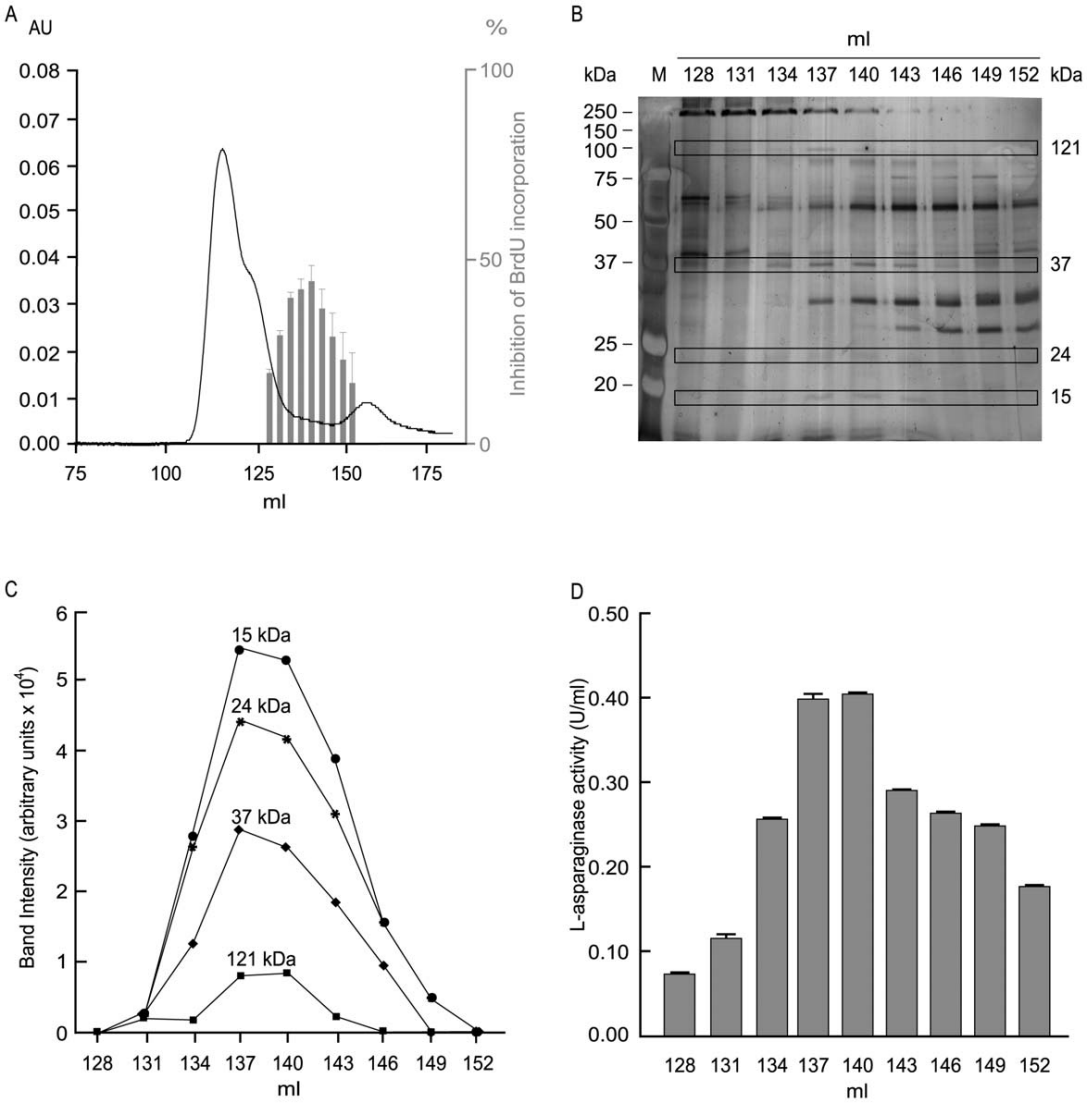
Isolation of the cell-cycle inhibiting activity from BCF. (A) Elution profile of BCF (black line) and cell-cycle inhibiting fractions identified by BrdU incorporation in HDF cells (grey histograms). (B) Active fractions separated on a non-reducing SDS gel. The four silver-stained bands of different molecular weights analysed by LC-MS/MS are in rectangles. (C) Stacked line representation of the relative intensities displayed by each of the bands detected in SDS-PAGE in (B). Numbers correspond to molecular masses in kDa. (D) L-asparaginase activity of cell-cycle inhibiting fractions.

BCF from *H. pylori* CCUG 17874 typically inhibited 5-bromo-2′-deoxy-uridine (BrdU) incorporation of normal human diploid cells (HDF) by 39.9±23.1% (n = 61, P<10^−27^). A typical elution profile of BCF obtained by size-exclusion chromatography on a Hi-Load Superdex 75 column is represented in Fig. 1A (black line). Active fractions inhibited BrdU incorporation by HDF cells (Fig. 1A, grey histograms) and showed compromised BrdU incorporation between 15.0±8.1 and 41.6±4.0% versus untreated control (100% BrdU incorporation). Separation of active fractions on a non-reducing sodium dodecyl sulphate (SDS)-PAGE (Fig. 1B) gave only 4 silver-stained bands of different molecular masses with an intensity profile matching the profile of cell-cycle inhibiting activity (Fig. 1C and Supplementary Fig. S1). Analysis of these bands by LC-MS and MS-MS revealed multiple proteins (Table 1), with their N-terminal sequences confirmed in several cases (data not shown). With the exception of γ-glutamyltranspeptidase (GGT) and L-asparaginase, detected in the bands corresponding to approximately 15 and 121 kDa, respectively, no other homologues of the other proteins found have been reported to interfere with the cell cycle. GGT (locus tags HP1118 in the *H. pylori* 26695 genome) is responsible of cell-cycle inhibition in T-cells [24] and in AGS cells [25]. The expected molecular mass of this protein in native conditions is 60 kDa, but a fragment with a molecular mass of 13.3 kDa and corresponding to residues 448–564 was previously isolated by 2D electrophoresis and peptide fingerprinting in the *H. pylori* secretome [26]. Our MS data are compatible with the predicted tryptic peptides derived from this fragment (49.6% coverage) and indicate that the latter is likely to exist as an independent unit whose significance is thus far unknown. L-asparaginase (locus tag HP0723, “probable asparaginase by sequence similarity”), belongs to an important family of related amidohydrolases that catalyse the deamidation of L-asparagine (Asn) [27]. The molecular mass of a single subunit is 37 kDa, but, being in the absence of denaturation, the enzyme was correctly detected in its tetrameric functional form (theoretical MW 144 kDa). Cells lacking a functional asparagine synthetase and exposed to L-asparaginase undergo cell-cycle arrest in G1 and, in some cases, such as Acute Lymphatic Leukemia (ALL), die by apoptosis [28]. These features prompted us to investigate whether GGT and L-asparaginase were involved in this process.

**Table 1:** Mass spectrometry data.

MW ×10^3^	Locus tag	Protein name
121	HP0723HP0875HP0011jhp11044jhp1290	L-AsparaginaseCatalaseChaperoninPurine nucleoside phosphorylase deoD-type(3R)-hydroxymyristoyl-[acyl-carrier-protein] dehydratase
37	HP1563HP0736HP0011jhp0098jhp0673	PeroxiredoxinAminotransferaseChaperoninCystathionine gamma-synthaseAminotransferase
24	HP1178	Purine nucleoside phosphorylase deoD-type
15	HP1118	Gamma-glutamiltranspeptidase fragment

### GGT is not responsible for HDF cell-cycle inhibition

**Figure 2:**
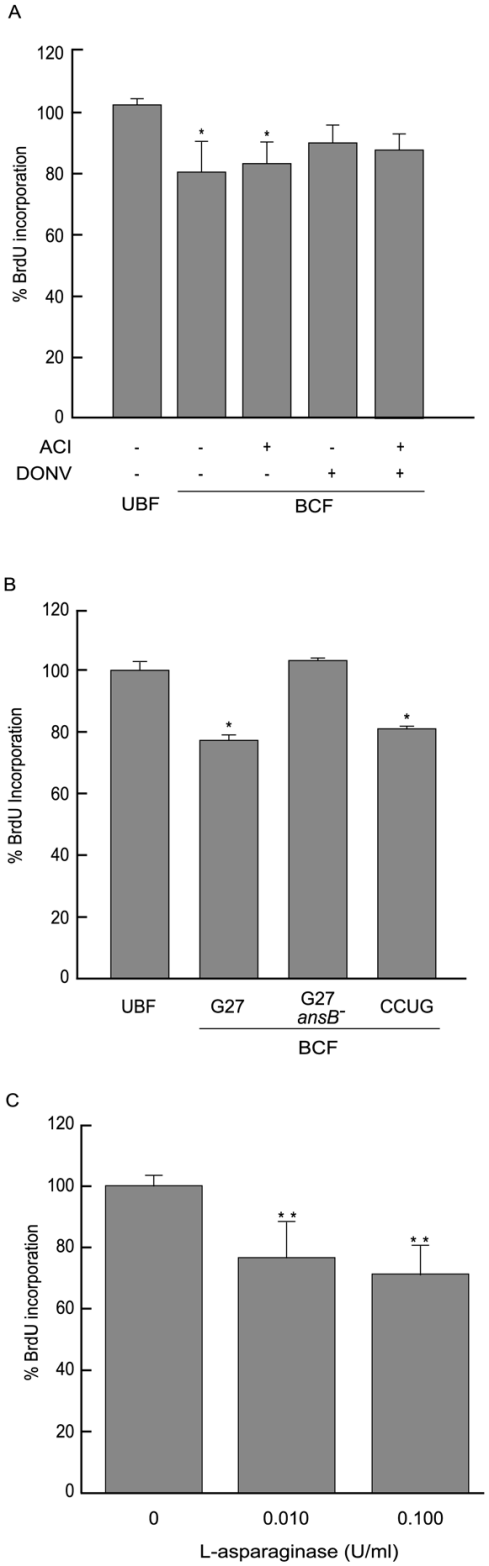
Cell-cycle inhibition in HDF cells. BrdU incorporation in HDF cells incubated with: (A) UBF and BCF treated with the L-asparaginase inhibitor 5-diazo-4-oxo-L-norvaline (DONV, 20 mM) or the GGT inhibitor acivicin (ACI, 5 mM), (B) UBF and BCF derived from different *H. pylori* strains, (C) different concentrations of recombinant L-asparaginase. Results are expressed as a mean ± SD from 5 independent experiments. ^*^P≤0.05, ^**^P≤0.01.

GGT activity of BCF was approximately 0.006 U/ml and was totally inhibited by the GGT specific inhibitor acivicin (ACI) (Table 2). Despite this, the capability of BCF to affect BrdU incorporation by HDF cells following GGT inhibition was unaltered (Fig. 2A, P<0.05 versus uninoculated broth culture filtrate, UBF). This demonstrated that GGT was not involved in the effect on the cell cycle we observed. The disagreement between the results reported in T-lymphocytes and AGS cells [24], [25] on one side, and our data on the other underline the importance of the specific cell lineage investigated and suggests that a single bacterial factor can have different effects on different cells types. Also, the lack of inhibition by DON, a well known inhibitor of GGT activity along with acivicin, might indicate a specific feature of the catalytic site. GGT activity of BCF derived from both G27 and G27 *ansB* ^-^ strains averaged at 0.0005 U/ml.

**Table 2:** GGT and L-asparaginase activity.

	BCF	ACI	DON	DONV
**GGT (U/ml)**	0.006	0.000*	0.006	N.D.
**L-asparaginase (U/ml)**	0.021	0.019	0.016	0.005*

BCF: Broth Culture Filtrate; ACI: acivicin; DON: 6-diazo-5-oxo-L-norleucine DONV: 5-diazo-4-oxo-L-norvaline; GGT: γ-glutamyltranspeptidase; SD≤11%; *: P<0.05 versus BCF.

### L-asparaginase activity is a common feature of *H. pylori* strains

Chromatographic fractions with cell-cycle inhibiting activity showed an L-asparaginase activity ranging from 0.075 to 0.4 U/ml, (Fig. 1D), and showed a profile consistent with that observed for cell-cycle inhibition (Fig. 1A). BCF produced with the CCUG 17874 strain possessed an L-asparaginase activity with a typical activity of 0.021 U/ml. This was found to be a common feature of *H. pylori* strains: the measured activity was approximately 0.100 U/ml for BCF derived from the G27 strain, 0.029 U/ml for the G21 strain and 0.027 U/ml for the 60190 strain.

### The specific inhibitor DONV inhibits L-asparaginase activity and affects BCF inhibition of the cell cycle

An L-asparagine mimic (5-diazo-4-oxo-L-norvaline, DONV), and the diazo analogue of L-glutamic acid 6-diazo-5-oxo-L-norleucine (DON) are known to act as specific suicidal inhibitors of glutaminase-asparaginases, and have been used in a variety of applications including the labelling of active sites [29]. BCF exposed to DONV showed an average reduction of L-asparaginase activity of 76.2% (0.005 U/ml versus 0.021 U/ml, Table 2) and BrdU incorporation of HDF cells incubated with DONV-treated BCF returned to values comparable to the control (Fig. 2A, P>0.05). A similar result was observed with BCF pre-incubated with both DONV and acivicin (Fig. 2A, P>0.05). Concentrations of DONV similar to those expected after its removal by diafiltration did not interfere with the cell cycle (data not shown). DON caused no significant reduction of L-asparaginase activity (0.016 U/ml versus 0.0021 U/ml, Table 2). These results demonstrate that L-asparaginase is involved in cell-cycle inhibition induced in HDF cells by *H. pylori* BCF. Furthermore, the differential effect of the inhibitors DON and DONV suggests a catalytic site with a strong preference for L-asparagine rather than L-glutamine.

### BCF from a G27 ansB^-^ mutant lacks L-asparaginase activity and displays reduced cell-cycle inhibiting activity

Mutagenesis experiments aimed at producing CCUG 17874 strains lacking L-asparaginase were unsuccessful. As an alternative, a mutant was generated in the G27 strain whose *ansB* gene (HPG27_679) had been previously isolated and which shows close homology with the CCUG 17874 *ansB* gene (HP0723) [30] (Supplementary Fig. S2). BCF derived from the *ansB^-^* G27 strain had no L-asparaginase activity (0.0±0.001 U/ml) and showed a BrdU incorporation similar to the control (P>0.05), while the wild-type G27 and CCUG strains had a BrdU incorporation reduced by 22.7±4.2% and 19.0±3.1%, respectively (both with P<0.05 versus UBF, Fig. 2B). Thus, the G27 knockout strain confirmed the loss of both the L-asparaginase and the cell-cycle inhibiting activity by the corresponding BCF.

### Cells sensitive to BCF are also sensitive to recombinant *H. pylori* L-asparaginase

In a previous study we described the production, biochemical characterisation and cytotoxicity of recombinant *H. pylori* L-asparaginase [31]. Now, we report that the recombinant enzyme, a type II L-asparaginase [32], [33], shows a strong preference for L-asparagine over L-glutamine, with a broad pH optimum activity in the range 7.0–10.0 and a drop of 80% activity below pH 4.0 [31] The *H. pylori* enzyme displayed a cytotoxic activity towards HDF cells and several tumour cell lines consistently higher than the *E. coli* orthologue, affecting survival in a concentration and cell-type dependent manner [31].

**Figure 3:**
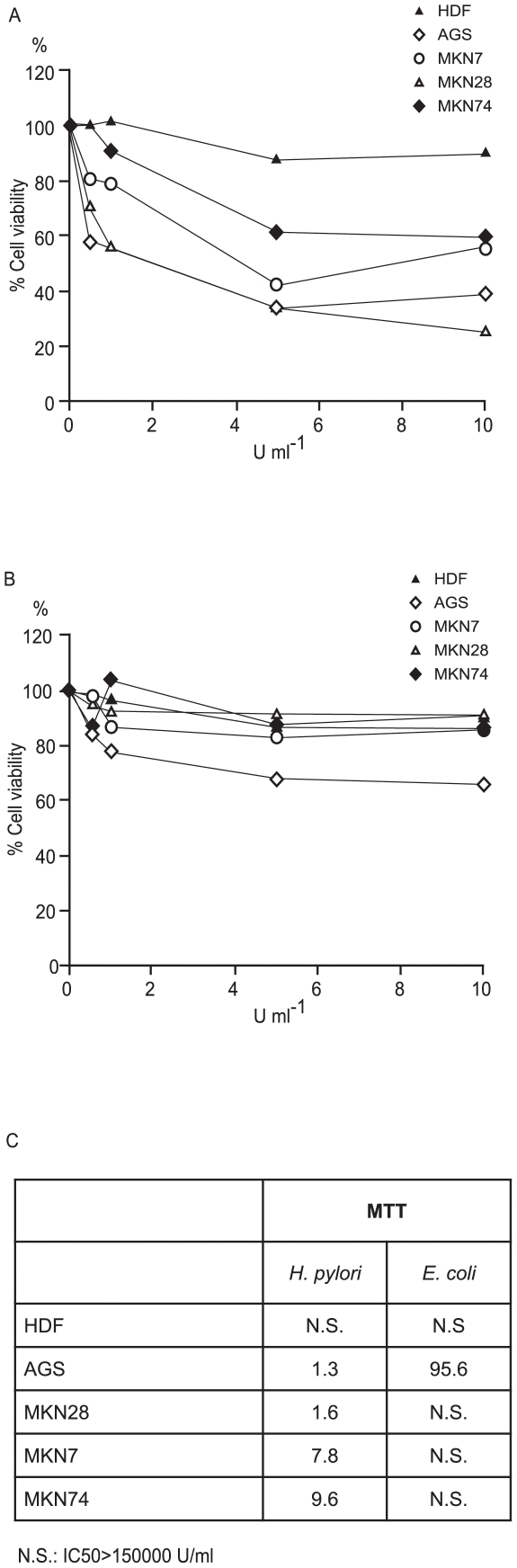
Cell-cycle inhibition by L-asparaginase. BrdU incorporation in cell lines exposed to variable concentration of recombinant *H. pylori* CCUG 17874 (A) and *E. coli* L-asparaginase (B) compared to untreated control cells. Boxes: magnification of the corresponding chart for L-asparaginase activities lower than 0.5 U ml^−1^. The points represent means (n≥3); SD not represented for clarity. (C) IC50 (L-asparaginase concentration inducing 50% cytotoxicity in U ml^−1^) of *H. pylori* and *E. coli* L-asparaginase (MTT assay).

Along these lines, we initially investigated the inhibitory activity of recombinant L-aparaginase on cell-cycle, monitoring BrdU incorporation into HDF cells at different enzyme concentrations. The enzyme caused an inhibition of cell-cycle of 23.4±13.8% at 0.01 U/ml and 28.9±9.9% at 0.1 U/ml (n≥3, P<0.01) (Fig. 2C), which confirmed that more than 50% of the effect exerted by BCF (39.9±23.1%, range: 16.8–63.0%) could indeed be ascribed to L-asparaginase. We then investigated the activity of the recombinant *H. pylori* enzyme on the cell-cycle of a group of tumoral gastric cell lines with different levels of differentiation (AGS, MKN28, MKN74, MKN7) and compared the effects with those exerted by the *E. coli* enzyme (Fig. 3A and 3B, respectively). All the cell lines exposed to *H. pylori* L-asparaginase showed a stronger cell-cycle inhibition than when exposed to comparable concentrations of the *E. coli* enzyme (Fig. 3C), with MKN7 and MKN28 displaying an IC_50_ difference of nearly 2 orders of magnitude. MKN74 was the less affected by both enzymes.

### Cell-cycle inhibition induced by L-asparaginase is related to the expression levels of asparagine synthetase

**Figure 4:**
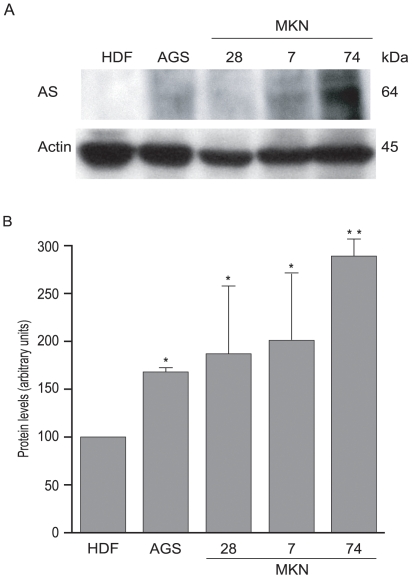
Expression of asparagine synthetase in cultured cell lines. (A) Western blot analysis of HDF, AGS, MKN28, MKN7 and MKN74 cell lysates for asparagine synthetase (AS). Actin was determined as loading control. (B) Densitometric analysis of protein levels normalized to the internal loading control. Results are expressed as a mean ± SD from 3 independent experiments ^*^P≤0.05, ^**^P≤0.01.

We then evaluated the relationship between asparagine synthetase expression and cell-cycle inhibition. For this, a Western blot analysis was performed for asparagine synthetase in the cell lines under examination (Fig. 4A). AGS, MKN7 and MKN28 cells expressed twice the amount, and MKN74 cells 3 times the amount of asparagine synthetase with respect to HDF cells (Fig. 4B). These expression levels were inversely related to cell-cycle inhibition induced by BCF [21] and by both *E. coli* and *H. pylori* recombinant L-asparaginases (Fig. 3C), with the notable exception of MKN7 and MKN28 for *H. pylori* L-asparaginase. While the former correlation is well documented, the latter exception might be related to their different genotype (Supplementary Table S2).

### Sub-cellular localisation of L-asparaginase in *H. pylori* CCUG 17874

**Figure 5:**
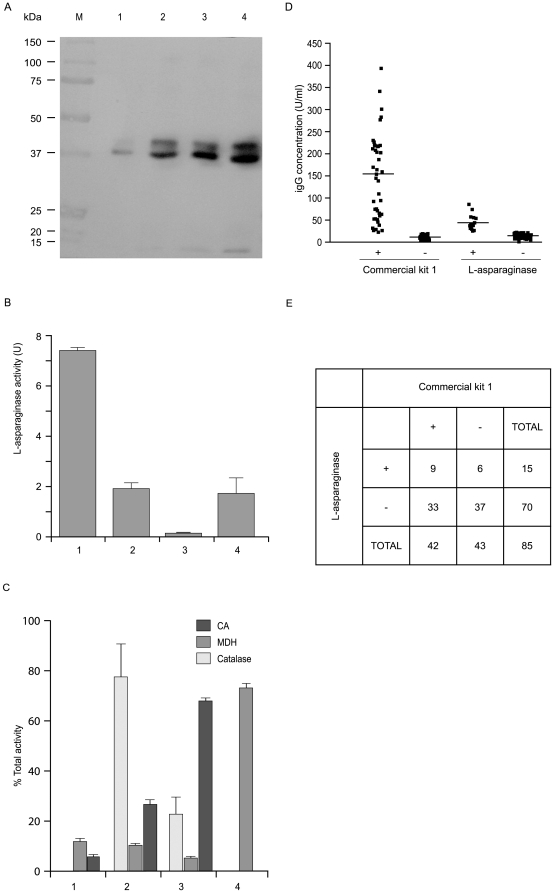
Subcellular localisation of L-asparaginase and ELISA assays. (A) Western blot performed on 100 µg of the subcellular fractions of *H. pylori* CCUG 17874 with an anti-asparaginase antibody. From left to right: 1: pre-lytic fraction; 2: periplasmic fraction; 3 and 4: soluble and insoluble spheroplast fractions, respectively. Molecular mass markers (kDa) are indicated. (B) Total L-asparaginase activity associated with each fraction (U). (C) Catalase, carbonic anhydrase (CA) and malonate dehydrogenase (MDH) were found in the expected *H. pylori* subcellular fractions: periplasm (2)[34], spheroplast (3) [35] and cytoplasm (4) [36], respectively. (D) Scatter-plot of ELISA assay results on sera from 42 *H. pylori* positive and 43 *H. pylori* negative patients. Serum samples (1∶101 dilution) were tested in parallel using commercial kit 1 and plates pre-coated with recombinant *H. pylori* L-asparaginase. Medians indicated as horizontal bars. 21% of the total samples were anti-L-asparaginase IgG positive (+). (E) Comparison of the results obtained with commercial kit 1 and L-asparaginase-based ELISA.

Sub-cellular fractions of *H. pylori* were prepared as reported in the Materials and Methods section. The purity of subcellular fractions was assessed by measuring the activities of marker enzymes. Catalase, carbonic anhydrase (CA) and malonate dehydrogenase (MDH) were found in the expected *H. pylori* subcellular fractions: periplasm [34], spheroplast [35] and cytoplasm [36], respectively (Fig. 5C). As expected, no activity of CA was found in the pre-lytic fraction. A significant level of cross-contamination was only observed between the periplasmic and spheroplast soluble fraction (about 25%, 2 and 3 in Fig. 5C), which means that L-asparaginase amounts detected in the corresponding fractions might differ by this percentage. Equivalent amounts of proteins from all the subcellular fractions were analysed by Western blot to localise L-asparaginase. The total L-asparaginase activity of each fraction was also determined. Two L-asparaginase forms with different molecular masses were identified in the periplasm and in the spheroplast soluble and insoluble fractions (Fig. 5A, lanes 2, 3 and 4). The higher molecular mass form (approximately 39 kDa) was less represented (40%) than the lower one (about 37 kDa, 60%). The 2 kDa difference between the two agrees with the approximate molecular mass calculated for the cleavable signal sequence predicted by the programs SignalP 3.0 [37] and PSORTb [38]. Interestingly, the pre-lytic fraction included mainly the 37 kDa-form (Fig. 5A, lane 1), suggesting that, in appropriate conditions, this form of the enzyme is free to leak through the outer bacterial membrane, while the 39 kDa-form might be membrane associated, as predicted by the BII server (http://protein.bii.a-star.edu.sg/sgi-bin/localization/gram-negative). It is also worthwhile noting that enzyme activity showed a progressive increase going from the cytoplasm to the periplasmic space (Fig. 5B). This supports the hypothesis of a maturation of the enzyme during its progress towards the latter. All this evidence, along with its presence in BCF, suggests that L-asparaginase can reach the external microenvironment, and therefore the host, in a process likely to involve a secretion system.

### ELISA assays

To determine whether *H. pylori* L-asparaginase is immunogenic in humans, we developed a *H. pylori* specific ELISA assay. Nearly 21% (9/43) of patients positive on the *Helicobacter pylori* IgG test (commercial kit 1, gold standard for the identification of previous or ongoing bacterial infection) were also positive for anti-L-asparaginase IgG, thus indicating that the protein causes an immune response in a subset of hosts (Fig. 5D). We found a lower IgG concentration for L-asparaginase as compared to commercial kit 1 (44.8±17.5 versus 148.5±95.5 U/ml) (Fig. 5D) and suspect that this is due to the multiple antigens used in the latter. Intriguingly, we found that 6 out of 43 patients negative by commercial kit 1 (14%) were positive for the L-asparaginase-based ELISA (Fig. 5E). This was not due to cross-reactivity since none of these patients were positive to *E. coli* L-asparaginase. Moreover, all of them were positive for *H. pylori* when commercial kit 2 was employed, suggesting that the antibodies detected were specific for *H. pylori* L-asparaginase and that this novel antigen may have a potential diagnostic application. Future work in our laboratory will seek to clarify this.

## Discussion

This work builds on our previous findings [21] on the cell-cycle inhibiting effect of *H. pylori* BCF on HDF and epithelial cell lines derived from human gastric tumours with different levels of differentiation (AGS, MKN28, MKN74 and MKN7), and focuses on the isolation and characterisation of L-asparaginase as the factor responsible for cell-cycle inhibition.

L-asparaginases (EC 3.5.1.1) are amidohydrolases that primarily catalyse the conversion of L-asparagine to L-aspartate and ammonia. Bacterial asparaginases are 140–150 kDa tetramers [39] built of identical subunits of 300–350 amino acid residues each, and include four independent catalytic sites [39], [40]. Some of them, especially the *E. coli* enzyme, display anti-leukemic activity, which has been primarily attributed to the reduction of L-asparagine in blood [41], [42].

Our genetic and functional characterisation of *H. pylori* CCUG 17874 L-asparaginase [31] led us to identify that the *ansB* gene, which consists of 993 bp (330 residues), shows a good homology to other *H. pylori* strains at the nucleotide, amino acid sequence and secondary structure level (Supplementary Fig. S2). As the activity of the recombinant enzyme steadily increases along the pH gradient observed throughout the gastric mucus layer (2.25–6.96) [43], a peak of activity is likely to occur *in vivo* at the epithelial surface. In this respect, previous work has showed that L-asparaginase is, in fact, an acid-inducible gene [30] and a virulence factor [44].

Following exposure to *H. pylori* BCF, HDF, MKN7 and MKN28 cells showed a significant increase in the number of cells in G1 phase, and, concomitantly, a decrease in those in the S phase [21]. In AGS cells, BCF induced a modest increase in the number of cells in G1 phase (by about 16%), without any significant decrement in S phase compared to controls [21]. No effect was observed on the cell cycle of the MKN74 cell line [21]. Similar changes in BrdU incorporation were observed when purified L-asparaginase was examined. In all cases, the *E. coli* enzyme appeared to be less active than the *H. pylori* one.

Previous studies have shown that treatment of leukemic cells with *E. coli* L-asparaginase causes arrest in the G1 phase [28], [45] and triggers the apoptotic pathway. However, L-asparaginase may also cause toxicity without inducing programmed cell death [46]–[49]. The factors leading to these alternative effects are not yet known. The mechanism of action of bacterial L-asparaginases seems to depend on their capability to reduce the levels of L-asparagine, for which some cell-types are auxotrophes. In fact, the cell-cycle block of cell clones, especially leukemic, exposed to this enzyme class has been previously described to depend on the expression level of asparagine synthetase [50]. A similar correlation is present in the cell lines we tested to recombinant *H. pylori* L-asparaginase. Interestingly, MKN7 and MKN28 cells represent an unexpected exception: they express relatively high levels of asparagine synthetase but at the same time their cell-cycle is strongly inhibited by *H. pylori* L-asparaginase. High expression of asparagine synthetase might be related to their carrying a mutant p53, a possible transactivator of the asparagine synthetase gene (http//atlasgeneticsoncology.org/Genes/ASNSID44323ch7q21.html), while the relatively high sensitivity of their cell-cycle to L-asparaginase might depend on the lack of p21^WAF1/CIP1^, that might thus act as a resistance barrier. p15 and p16, absent in MKN28 but present in MKN7, do not seem to significantly affect BrdU incorporation. Cyclin E is absent in MKN74 cells and, interestingly, proliferation of these cells is not inhibited by BCF or L-asparaginase. All this evidence suggests that cell-cycle proteins might have a pivotal role in mediating L-asparaginase effects and suggest the need for further investigations.

Along this line, we speculated that a different status of the L-asparagine synthesis pathway in different cell types of the gastric epithelium could condition their response to bacterial L-asparaginase. In fact, preliminary immunohistochemistry data of human acido-peptic mucosa demonstrate an unequal expression of asparagine synthetase throughout the gastric epithelium, with a clear localisation at the base of the glands up to the level of mucous neck cells (Supplementary Fig. S3). Inhibition of cell-cycle of cells lacking asparagine synthetase could favour the proliferation of those carrying the enzyme, thus becoming more suitable to overcome the action of an L-asparagine deficiency. Thus, though the main mechanism of action of *H. pylori* L-asparaginase is expected to be its contribution to buffer the acidic microenvironment of the stomach through ammonia production, similarly to urease, during the process of *in vivo* colonisation, it could also interfere with the cell cycle of the epithelium altering the normal balance between proliferation and apoptosis.

Contact between L-asparaginase and the host requires the release or secretion of the enzyme to the external environment. In good agreement with this idea, our data show that the highest enzyme activity was detected in the most external subcellular bacterial fraction although its presence was ubiquitous. This, along with the observation that the protein has a predicted signal peptide, suggests that protein transit into the external environment might occur through secretion or through release of outer membrane vesicles [51], leading to immune stimulation of the host and the production of specific IgGs. We have demonstrated that these antibodies allow detection of infected patients otherwise undetectable by one of the available commercial kit.

In conclusion, the present work shows that *H. pylori* L-asparaginase is a relevant actor in cell-cycle inhibition and a promising diagnostic tool. Its role as a potential pathogenic factor will now be thoroughly analysed both by direct administration of the protein to animal models and by analysing its internalisation in cells of infected patients.

## Materials and Methods

### Ethics statement

At the time this study was performed, Institutional Ethical Boards in Italy were devoted only to the regulation, authorisation and monitoring of pharmaceutical studies and not of medical devices, including new reagents for *in vitro* diagnosis. Patients with gastric symptoms who had to undergo blood sample testing were however asked to sign a written informed consent form at the Centro Analisi Monza (CAM, Monza, Italy). Mice immunization was conducted according to the guidelines of the European Communities Directive 86/609/EEC regulating animal research and following a protocol approved by the Ministry of Health (n. 10169321466.9) in accordance with national laws.

### Bacterial strains and broth culture filtrate (BCF) production

We used urease positive, VacA^+^, CagA^+^ *H. pylori* strains CCUG 17874 (Culture Collection University of Goteborg, Goteborg, Sweden), 60190 (ATCC 49503), G21 and G27, and the urease-positive, VacA^-^, CagA^-^ G21 *H. pylori* strain (kindly provided by N. Figura, Siena, Italy).

For routine culture, the bacterial broth culture filtrate (BCF) and Uninoculated Broth culture Filtrate (UBF) were produced in brucella broth (Difco, Detroit, MI) supplemented with 5% inactivated foetal bovine serum (FBS, Invitrogen, Milan, Italy), Skirrow and Vitox (Oxoid, Basing-stoke, UK) as described [21]. For biochemical studies, the CCUG 17874 strain was initially cultured as above until the OD_450_ reached 1.0 Absorbance Units (AU). Brucella broth was gradually replaced by F10 (Invitrogen, Milan, Italy) by subsequent two-fold dilutions. The culture medium was then removed by centrifugation and bacteria resuspended in an equal volume of F10 supplemented with Skirrow, Vitox and 200 µg/ml β-cyclodextrin [52]. As the cell culture was viable for 7 d in these conditions, BCF was consistently prepared on day 6.

### Preparation of *H. pylori* G27 ΔHP0723::KanSacB, KanR, Sucrose Sensitive mutant

A G27 ΔHP0723::KanSacB, KanR, Sucrose Sensitive mutant was prepared using the primers of Supplementary Table S1. A portion of the N-terminal and C-terminal regions of the *ansB* gene (HPG27_679) were amplified and fused together using Splicing by Overlap Extension (SOE) PCR. This strategy deleted the internal portion of the gene and added an *Xho* I and *Sma* I site for cloning of the KanSacB cassette from pKSF-II. The SOE reaction was conducted in a series of 3 PCR reactions. In the first, the *ansB*-Forward 1 and *ansB*-Reverse 1 PCR primers were used to amplify the N-terminal region of *ansB* from G27. In the next round, *ansB*-Forward 2 and *ansB*-Reverse 2 were used to amplify the C-terminal region of *ansB*. These products were purified, mixed and the SOE reaction performed using the *ansB*-Forward 1 and *ansB*-Reverse 2 primers. This PCR product was then subcloned into pGEMT-Easy and the proper insert verified by *EcoR* I, *Xho* I and *Sma* I digestion. This plasmid was subsequently digested with *Xho* I and *Sma* I and the KanSacB fragment from pKSF-II, which was obtained by similar digestion, was ligated to the *ansB* deletion construct. The resulting verified plasmid was transformed into G27 and transformants were selected on Kan 25. The resulting isolate was analyzed with the *ansB*-Rev-Far downstream primer that lies outside of all of the primers used to make the mutation construct along with the sacBSCN-F2 primer to ensure proper insertion of the cassette into the chromosome. Additionally, the PCR product was sequenced.

### Cell lines and culture conditions

Normal human diploid embryonic fibroblasts (HDF, kindly donated by J. Sedivy, Brown University Providence, RI) and epithelial cell lines derived from human gastric tumours with different levels of differentiation (AGS [53], MKN28, MKN74, MKN7 [54]) were routinely grown at 37°C in E-MEM (HDF), RPMI 1640 (AGS) and DMEM/Ham's nutrient mixture F-12 (MKN7, MKN28, MKN74) containing 100 IU ml^−1^ penicillin and 100 µg ml^−1^ streptomycin, and supplemented with 10% FBS, in a 5% CO_2_ humidified atmosphere. Cells were used in exponential growth throughout all the experiments.

### 5-Bromo-2′-deoxy-Uridine assay

For routine assay of cell-cycle progression, cells were seeded in 96-well microtitre plates at 4×10^3^ cells/well in a final volume of 100 µl medium. After 24 h growth, treatments were performed on subconfluent cell monolayers in 150 µl volumes per well and incubated at 37°C for 24 h. Progression throughout the S phase was measured with the 5-Bromo-2′-deoxy-Uridine Labelling and Detection kit III (Roche Applied Biosciences, Mannheim, Germany) by incubating the samples in the presence of 10 µM 5-bromo-2′-deoxy-uridine (BrdU) for the last 2 h of treatment.

### Size exclusion chromatography

For isolation of the cell-cycle inhibiting factor, one litre of BCF derived from an *H. pylori* CCUG 17874 culture in F10 medium was concentrated to 10 ml with a 10 kDa cut-off concentrator and loaded onto a Hi-Load 26/60 Superdex 75 column (GE Healthcare Europe GmbH, Milan, Italy) equilibrated in 60 mM Tris-HCl, pH 8.0, 100 mM NaCl, using an Amersham FPLC system. Elution was performed at 3 ml/min with equilibration buffer and spectrophotometrically monitored at 280 nm. Three ml fractions were collected and assayed in triplicate for cell-cycle inhibiting activity using the BrdU assay. Twenty μl of each active fraction were analysed by a 12% SDS-PAGE after mixing with 10 µl 65 mM Tris-HCl, pH 7.5, 1% SDS, 10% glycerol, 0.02% bromophenol blue as a non-reducing buffer. To increase sensitivity, after Coomassie staining, the gel was silver stained as described by Hochstrasser [55].

### Liquid chromatography-mass-spectrometry (LC-MS) and MS-MS

Two hundred μl of each active fraction were concentrated 10 times using a 10 kDa cut-off concentrator, and loaded on a 10% SDS-PAGE in non-reducing conditions. After Coomassie-staining, bands whose intensity staining profiles matched the profile of cell-cycle inhibiting activity were cut from the gel and processed for subsequent trypsin digestion and liquid chromatography-mass-spectrometry (LC-MS/MS) analysis on a Q-ToF Ultima Global (Micromass, Waters), with an ESI source coupled with an online nano-HPLC [56]. The results were analysed using the Mascot software (http://www.matrixscience.com).

### Enzymatic assays

γ-glutamyltransferase (GGT) was assayed with an enzymatic colorimetric assay in which the enzyme transferred the glutamyl group of L-γ-glutamyl-3-carboxy-4-nitroanilide to glycyl-glycine [57]. One Unit is defined as the amount of enzyme that catalyzes the transformation of one micromole of substrate per min at 37°C. L-asparaginase activity was measured by a stopped assay using Nessler's reagent [27], or, alternatively, by the MAAT Asparaginase activity test (Medac, Wedel, Germany). One unit is the amount of enzyme catalysing the production of 1 µmol of ammonia per min at 37°C. The changes in the slope of time-dependent reduction in hydrogen peroxide absorbance at 240 nm (PBS, pH 7.2) were used as a measure of catalase activity and were calculated according to the method of Beers & Sizer [58]. One unit of catalase activity decomposes 1 mmol hydrogen peroxide at 240 nm per min at a substrate concentration of 10 mM. MDH and CA activities were assayed spectrophotometrically [59], [60]. For MDH, one unit is defined as the amount of enzyme that will oxidize 1.0 µmol NADH per min at 25°C, pH 7.5. For CA, one unit will cause the pH of a 0.012 M buffer to drop from 8.3 to 6.3 per minute at 0°C.

### Enzymatic inhibition assays

BCF was mixed with an equal volume of PBS (control) or of one of the following: 40 mM solutions in PBS of the specific asparaginase inhibitors 6-diazo-5-oxo-L-norleucine (DON, the diazo analogue of L-glutamic acid) or 5-diazo-4-oxo-L-norvaline (DONV, an asparagine analogue), kindly donated by Prof. Pizzorno (University of Yale), ora 10 mM solution in PBS of the specific GGT inhibitor acivicin. Samples were incubated at room temperature for 16 h. L-asparaginase activity was measured with the MAAT activity test. They were then dialysed by diafiltration in 10 kDa cut-off concentrators until the concentration of inhibitors' was <1 nM. Inhibition of BrdU incorporation was then evaluated in HDF cells.

### *H. pylori* fraction preparation

The cell pellet obtained from a *H. pylori* CCUG 17874 50 ml culture was washed in 10 ml PBS, resuspended in 1.5 ml of 10 mM Tris-HCl pH 7.6, 20% sucrose and, after a 5 min incubation on ice, mixed with 50 µl of 0.5 M EDTA, pH 8.0. After a 10 min incubation and subsequent centrifugation, the supernatant containing proteins that leaked from the cells or were located at or near the cell surface was collected (fraction 1 or pre-lytic), whereas cells were converted into spheroplasts by resuspending them in 1 ml cold distilled water and incubating on ice for 10 min. After centrifugation, the supernatant (periplasmic fraction, fraction 2) was collected, whereas the spheroplasts were resuspended in 500 µl of 1 M Tris- HCl, pH 7.6, incubated on ice for 30 min and centrifuged. The supernatant (spheroplast soluble fraction, fraction 3) was recovered and the pellet (spheroplast insoluble fraction, fraction 4) resuspended in 500 µl of 50 mM Tris-HCl, pH 8.0, 2 mM EDTA, 0.1 mM DTT, 5% (v/v) glycerol.

### Production of recombinant H. pylori CCUG 17874 L-asparaginase

Recombinant L-asparaginase from *H. pylori* CCUG 17874 was produced in *Escherichia coli* BL21(DE3) cells harbouring construct pDC1 as described [31]. Activity was determined as described above.

### Production of a monoclonal anti-L-asparaginase antibody

For antibody production, a 6-month-old mouse (C57/B) was immunised once with 30 µg of recombinant *H. pylori* CCUG 17874 L-asparaginase supplemented with Complete Freunds Adjuvant (Pierce, Rockford, IL), and twice with 20 µg of enzyme supplemented with Incomplete Freunds Adjuvant (Pierce, Rockford, IL) every 4 weeks. Antibody titre was analysed by direct enzyme linked immunosorbent assay (ELISA). Spleen B cells were fused with an equal number of NS0 mouse myeloma cells and, the following day, the hybridomas seeded in petri dishes in selective medium. An IgM antibody with good reactivity was then isolated from 1l of the culture supernatants using Protein A Sepharose (GE Healthcare, Europe GmbH, Milan, Italy) and subjected to biotinylation with the FluoReporter Mini-Biotin-XX Protein Labelling Kit (Molecular Probes, Invitrogen, Milan, Italy).

### Western Blotting

Protein samples and molecular weight markers (Precision Protein Standards, BioRad, Milan, Italy) were separated on a 12% SDS-PAGE in reducing conditions and transferred onto a polyvinylidene difluoride membrane (Millipore, Billerica, MA). The membrane was blocked with 5% (w/v) milk powder in PBS containing 0.05% Tween (PBS-T). For analysis of the subcellular localization of L-asparaginase, the membrane was incubated with the biotinylated mouse monoclonal anti-L-Asparaginase antibody at 1 µg/ml in PBS-T, 1% BSA for 1 h. Blots were washed 3 times (5 min each) in PBS-T, incubated with a streptavidin-horseradish peroxidase conjugate (GE Healthcare Europe GmbH, Milan, Italy) in PBS-T for 45 min, washed 3 times as above, and visualised by ECL (GE Healthcare Europe GmbH, Milan, Italy). For analysis of the expression levels of asparagine synthetase in mammalian cells, the membrane was incubated with a rabbit anti-asparagine synthetase polyclonal antibody (Epitomics, Burlingame, CA) at a 1∶5000 dilution in PBS-T, 1% BSA at room temperature for 1 h. After 3 washes in PBS-T (5 min each), the blot was incubated with a horseradish peroxidase conjugate anti-rabbit antibody (GE Healthcare Europe GmbH, Milan, Italy) in PBS-T for 45 min, washed 3 times, and visualised by ECL.

### Mammalian cell lysis

For Western Blotting, AGS, MKN7, MKN28, MKN74 and HDF cells, grown in 250 ml flasks, were lysed [61] and briefly sonicated in an ice-cold bath. The cell lysate was centrifuged at 12000 x g at 4°C for 30 min. Protein concentration of the supernatant was determined with the MicroBCA protein assay (Pierce, Rockford, IL). Aliquots containing 20 µg of proteins were utilised immediately.

### Enzyme-linked immunosorbent assay (ELISA)

In order to analyse patients' immune response to L-asparaginase, an ELISA assay was performed. Serum samples were collected and tested using a *Helicobacter pylori* IgG Enzyme Immunoassay test (commercial kit 1). 43 positive (IgG concentration>24 U/ml) and 42 negative (IgG concentration<20 U/ml) sera were then tested in parallel with both commercial kit 1 and an Immulon 96-well plate (Santa Cruz Biotechnology, Inc) pre-coated with *H. pylori* L-asparaginase at a final concentration of 1 µg/ml in PBS overnight. Samples which tested negative with the commercial kit and positive by L-asparaginase ELISA were re-assayed with a second commercial kit for *Helicobacter pylori* IgG (commercial kit 2).

### Statistical analysis

Student's *t* test was used for pairwise comparisons (n>10). Kruskal-Wallis non-parametric test followed by Bonferroni-type post-hoc analysis was used for multiple comparisons (n<10). Results are reported as mean±SD and P<0.05 was considered significant. IC_50_ (the L-asparaginase concentration inducing 50% cytotoxicity) values were determined with the software BioDataFit 1.02, available at the URL: http://www.changbioscience.com/stat/ec50.html

## Supporting Information

Figure S1 Band intensities. Three-dimensional histogram representation of the relative intensities displayed by each of the bands detected in cell-cycle inhibiting fractions by SDS-PAGE. On the x axis: molecular mass (kDa), on the y axis: relative intensity (arbitrary units), on the z axis: elution volume (ml).
(4.84 MB TIF)

Figure S2 L-asparaginase sequence alignment. (A) Amino acid sequence alignment of L-asparaginase from different strains of H. pylori: from top to bottom: strain CCUG 17874, J99, HPAG1 and G27. Black boxes include conserved regions, white boxes sequence differences. (B) A model of an L-asparaginase monomer, showing its conserved secondary structure.
(0.75 MB TIF)

Figure S3 Asparagine synthetase in the human stomach. (A) Asparagine synthetase distribution in human stomach mucosa at 4× magnification. Arrows indicate the basis of two positive glands. (B) Negative control. Paraffin embedded archive material was sectioned at 5 µm and stained with UltraVision LP Large Volume Detection System HRP Polymer and DAB Plus Chromogen (Thermo Fisher Scientific, Barrington, IL, USA). After deparaffinization and rehydration, tissue sections were washed twice in PBS and incubated in goat serum, washed 4 times in PBS and, to reduce nonspecific background staining due to endogenous peroxides, incubated in hydrogen peroxide for 10 min. Each washing step was performed in PBS for 5 min and repeated 4 times. After washing, Ultra V Block was applied and incubated at room temperature for 5 min. The slides were washed and incubated overnight at room temperature in buffer with (sample) or without (negative control) anti-Asparagine Synthetase Rabbit Monoclonal Antibody (Epitomics) diluted 1∶50. After washing, the Primary Antibody Enhancer was added and incubated at room temperature for 10 min. The slides were then washed and incubated in HRP Polymer at room temperature for 15 min, washed again and incubated with diaminobenzidine (DAB). Once rinsed 4 times in water, the slides were mounted with DPX and observed at an optical microscope (Eclipse, E400, Nikon).
(3.69 MB TIF)

Table S1 Primers for the preparation of H. pylori G27DeltaHP0723::KanSacB, KanR, Sucrose Sensitive mutant
(0.01 MB RTF)

Table S2 Expression of cell cycle proteins by cell lines used in the present study
(0.01 MB RTF)
