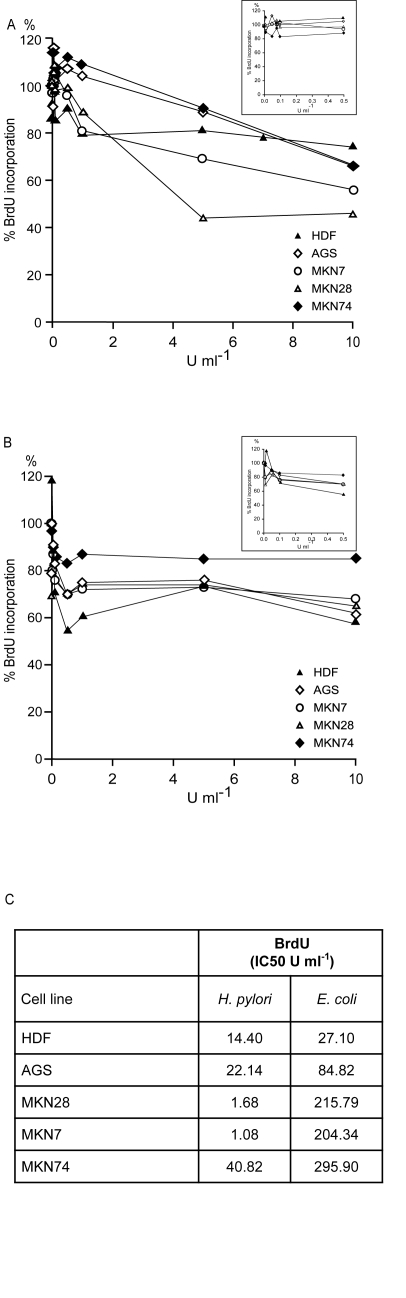# Correction: Cell-Cycle Inhibition by *Helicobacter pylori* L-Asparaginase

**DOI:** 10.1371/annotation/c6c16a43-aadd-4cd3-a916-e935534dab38

**Published:** 2012-02-23

**Authors:** Claudia Scotti, Patrizia Sommi, Maria Valentina Pasquetto, Donata Cappelletti, Simona Stivala, Paola Mignosi, Monica Savio, Laurent Roberto Chiarelli, Giovanna Valentini, Victor M. Bolanos-Garcia, Douglas Scott Merrell, Silvia Franchini, Maria Luisa Verona, Cristina Bolis, Enrico Solcia, Rachele Manca, Diego Franciotta, Andrea Casasco, Paola Filipazzi, Elisabetta Zardini, Vanio Vannini

Due to an error, a wrong image for Figure 3 was uploaded during the production process of this article; the uploaded figure included previous MTT data reported by the same group in Capelletti et al., BBRC, 2008; 377:1222-6. The peer review process for the manuscript was, however carried out with a version of the manuscript that included the correct Figure 3 reporting the BrdU data.

The authors are therefore issuing this Correction in order to make the Correct Figure 3 available to readers. The correct figure legend is as follows:

BrdU incorporation in cell lines exposed to variable concentration of recombinant H. pylori CCUG 17874 (A) and E. coli L-asparaginase (B) compared to untreated control cells. Boxes: magnification of the corresponding chart for L-asparaginase activities lower than 0.5 U ml-1. The points represent means (n ≥ 3); SD not represented for clarity. (C) IC50 (concentration in U ml-1 inducing 50% reduction of BrdU incorporation) of H. pylori and E. coli L-asparaginase.

The correct Figure 3 can be viewed here: 

**Figure:**