# Supplementary figures and images for: Cell-Cycle Inhibition by Helicobacter pylori L-Asparaginase
Source: PLoS One. 2010 Nov 9;5(11):e13892. doi: 10.1371/journal.pone.0013892 (PMC2976697; doi:10.1371/journal.pone.0013892)

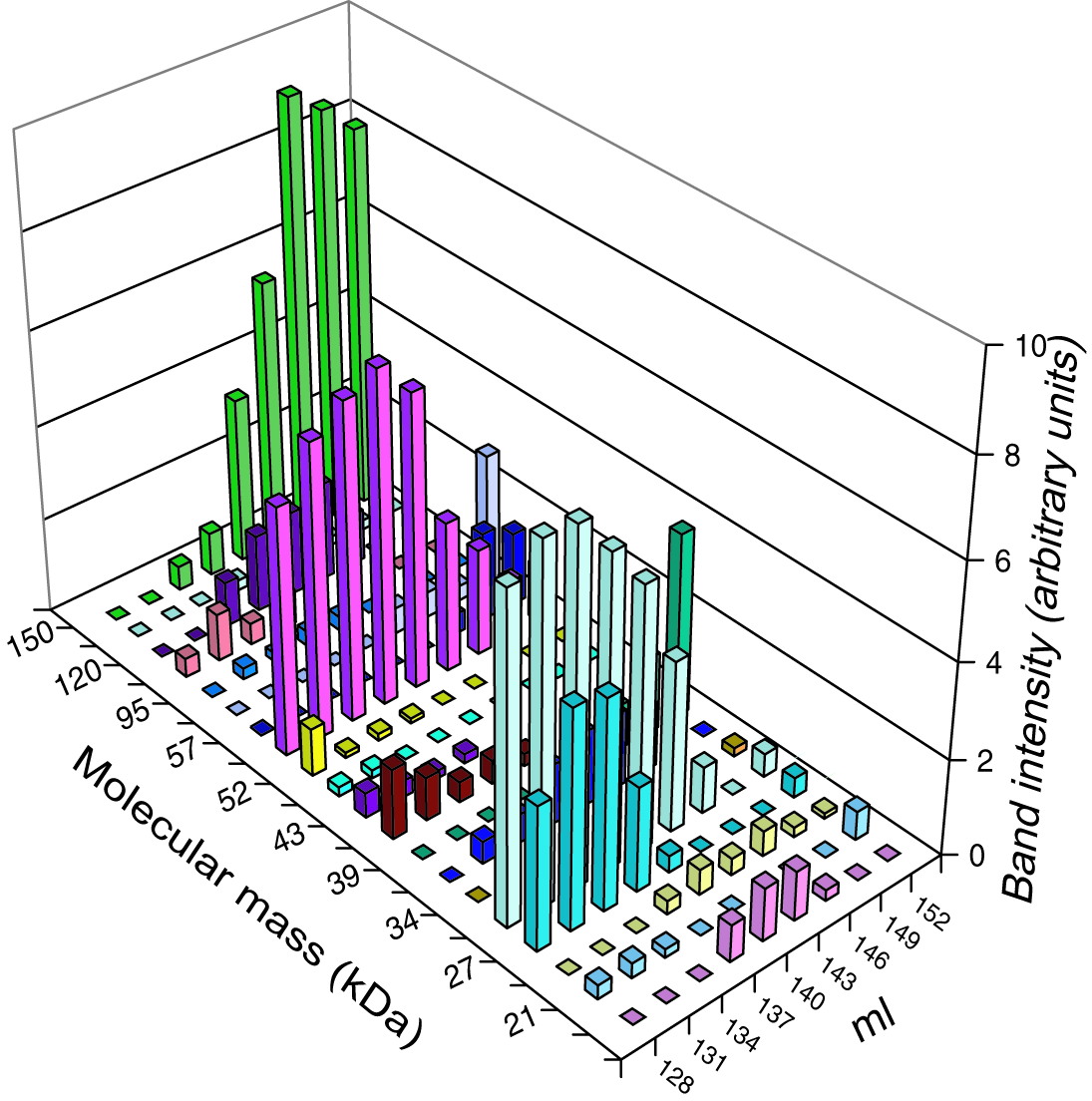

Supplement: Figure S1 — Band intensities. Three-dimensional histogram representation of the relative intensities displayed by each of the bands detected in cell-cycle inhibiting fractions by SDS-PAGE. On the x axis: molecular mass (kDa), on the y axis: relative intensity (arbitrary units), on the z axis: elution volume (ml). (4.84 MB TIF) [file pone.0013892.s001.tif]

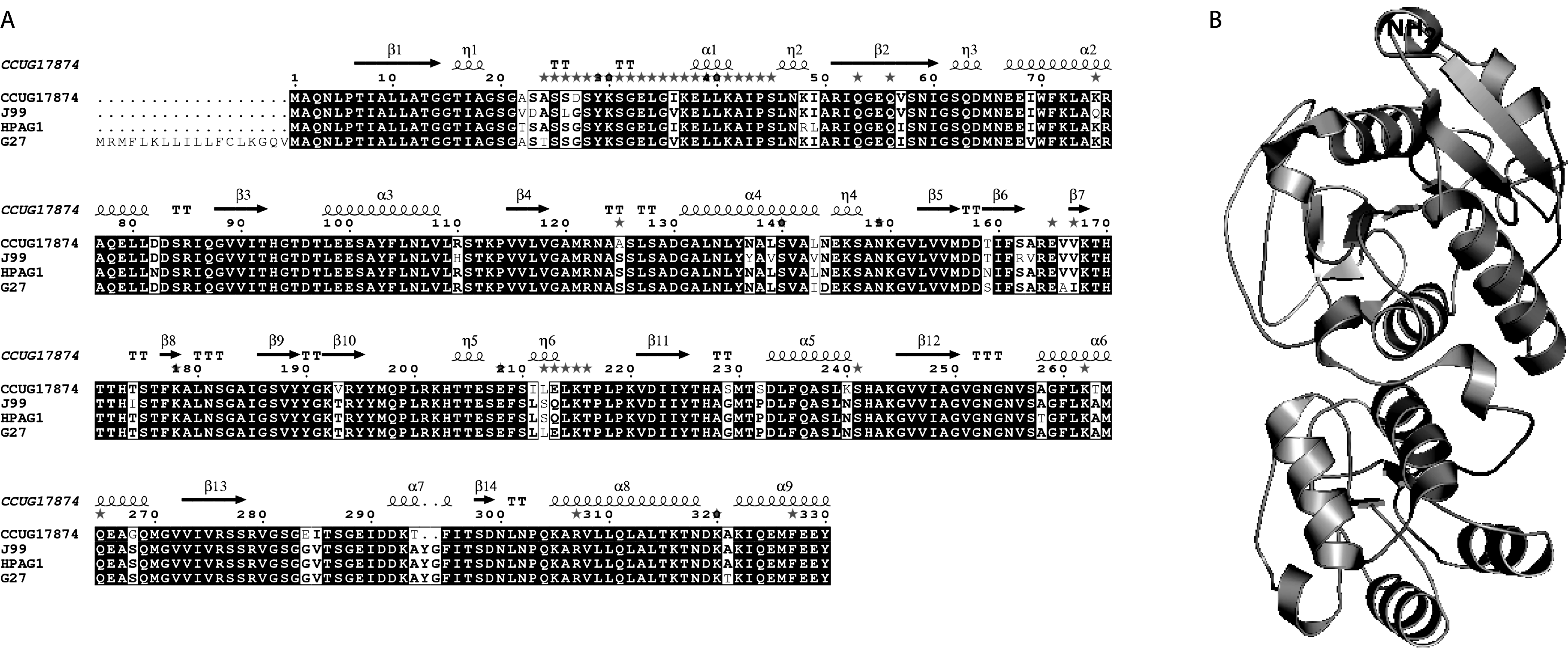

Supplement: Figure S2 — L-asparaginase sequence alignment. (A) Amino acid sequence alignment of L-asparaginase from different strains of H. pylori: from top to bottom: strain CCUG 17874, J99, HPAG1 and G27. Black boxes include conserved regions, white boxes sequence differences. (B) A model of an L-asparaginase monomer, showing its conserved secondary structure. (0.75 MB TIF) [file pone.0013892.s002.tif]

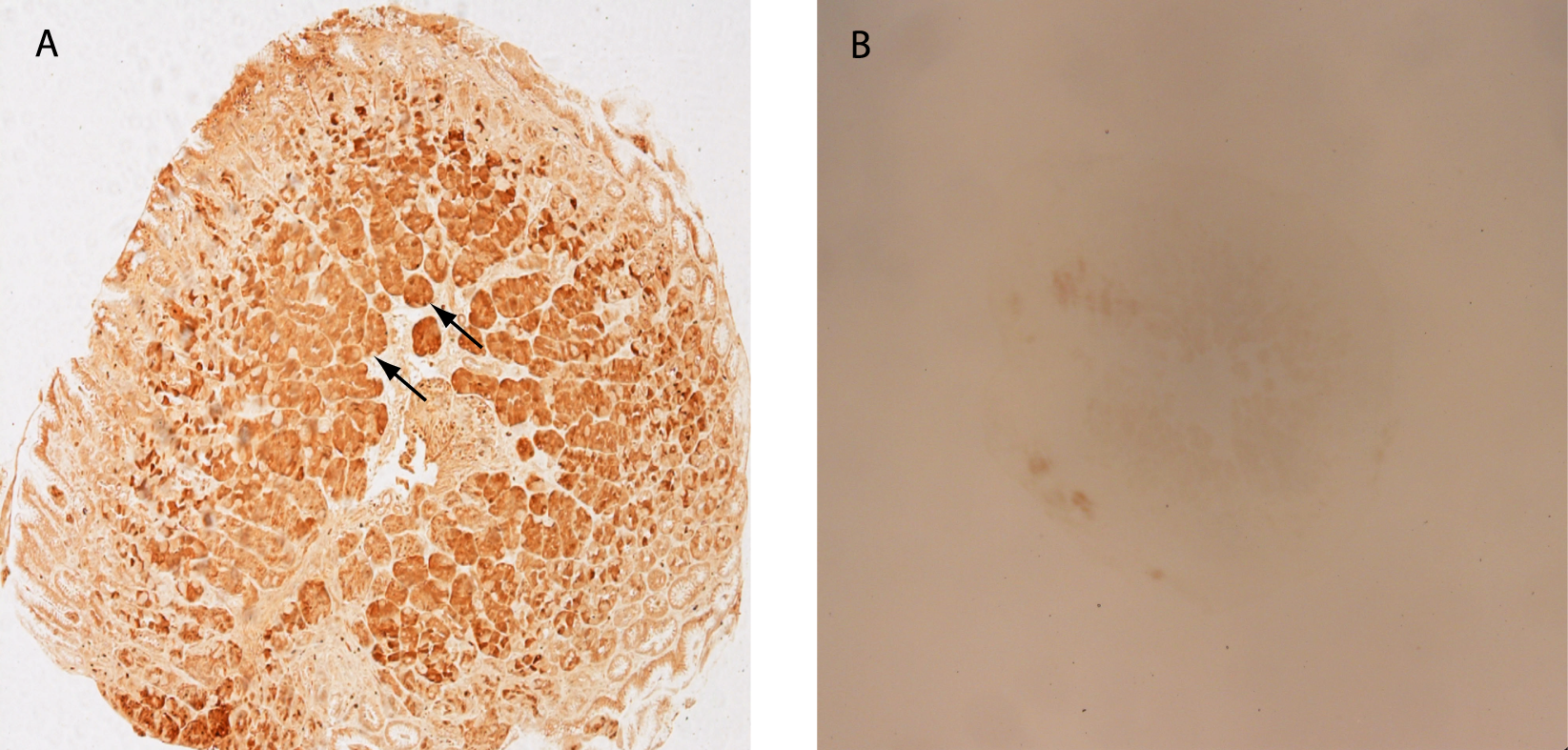

Supplement: Figure S3 — Asparagine synthetase in the human stomach. (A) Asparagine synthetase distribution in human stomach mucosa at 4× magnification. Arrows indicate the basis of two positive glands. (B) Negative control. Paraffin embedded archive material was sectioned at 5 µm and stained with UltraVision LP Large Volume Detection System HRP Polymer and DAB Plus Chromogen (Thermo Fisher Scientific, Barrington, IL, USA). After deparaffinization and rehydration, tissue sections were washed twice in PBS and incubated in goat serum, washed 4 times in PBS and, to reduce nonspecific background staining due to endogenous peroxides, incubated in hydrogen peroxide for 10 min. Each washing step was performed in PBS for 5 min and repeated 4 times. After washing, Ultra V Block was applied and incubated at room temperature for 5 min. The slides were washed and incubated overnight at room temperature in buffer with (sample) or without (negative control) anti-Asparagine Synthetase Rabbit Monoclonal Antibody (Epitomics) diluted 1∶50. After washing, the Primary Antibody Enhancer was added and incubated at room temperature for 10 min. The slides were then washed and incubated in HRP Polymer at room temperature for 15 min, washed again and incubated with diaminobenzidine (DAB). Once rinsed 4 times in water, the slides were mounted with DPX and observed at an optical microscope (Eclipse, E400, Nikon). (3.69 MB TIF) [file pone.0013892.s003.tif]
