# Supplementary material for: Cell-Cycle Inhibition by Helicobacter pylori L-Asparaginase
Source: PLoS One. 2010 Nov 9;5(11):e13892. doi: 10.1371/journal.pone.0013892 (PMC2976697; doi:10.1371/journal.pone.0013892)
Supplement: Table S1 — Primers for the preparation of H. pylori G27DeltaHP0723::KanSacB, KanR, Sucrose Sensitive mutant (0.01 MB RTF) [file pone.0013892.s004.rtf]

Table S1 Primers for the preparation of H. pylori G27 DHP0723::KanSacB, KanR, Sucrose Sensitive mutant

Primer	Sequence (5' to 3')	
ansB-Forward1	CTTTTAAATTTCAAAAGGTGGTCCATAATG	
ansB-Reverse1	CCCACCCCGGCTATCACAACGCCcccgggaggctcgagCGTTTAGCGAGCTTGAACCAC	
ansB-Forward2 	GTGGTTCAAGCTCGCTAAACGctcgagcctcccgggGGCGTTGTGATAGCCGGGGTGGG	
ansB-Reverse 2	CAAAATAAACCAAATCACCCAATTATC	
ansB-Rev-Far	GCGTGTATCTCAATTATGTGTTCGC	
sacBSCN-F2	CGAATCGAATTCAGGAAC	

 
