# Supplementary material for: Cell-Cycle Inhibition by Helicobacter pylori L-Asparaginase
Source: PLoS One. 2010 Nov 9;5(11):e13892. doi: 10.1371/journal.pone.0013892 (PMC2976697; doi:10.1371/journal.pone.0013892)
Supplement: Table S2 — Expression of cell cycle proteins by cell lines used in the present study (0.01 MB RTF) [file pone.0013892.s005.rtf]

Table S2. Expression of cell cycle proteins by cell lines used in the present study 

	p15	p16	p21 WAF1/CIP1	p27 KIP1	Cyclin A	Cyclin D1	Cyclin E	p53	
MKN28	-	-	-	+	+	+	+	mut	
MKN7	+	+	-	+	+	+	+	mut	
MKN74	-	-	+	+	+	+	-	wt	
AGS	?	?	+	+	+	+	+	wt	
HDF	+	+	+	+	+	+	+	wt	
